# Minimal changes in health status questionnaires: distinction between minimally detectable change and minimally important change

**DOI:** 10.1186/1477-7525-4-54

**Published:** 2006-08-22

**Authors:** Henrica C de Vet, Caroline B Terwee, Raymond W Ostelo, Heleen Beckerman, Dirk L Knol, Lex M Bouter

**Affiliations:** 1 EMGO Institute, VU University Medical Center, Van der Boechorststraat 7, 1081 BT Amsterdam, The Netherlands

## Abstract

Changes in scores on health status questionnaires are difficult to interpret. Several methods to determine minimally important changes (MICs) have been proposed which can broadly be divided in distribution-based and anchor-based methods. Comparisons of these methods have led to insight into essential differences between these approaches. Some authors have tried to come to a uniform measure for the MIC, such as 0.5 standard deviation and the value of one standard error of measurement (SEM). Others have emphasized the diversity of MIC values, depending on the type of anchor, the definition of minimal importance on the anchor, and characteristics of the disease under study. A closer look makes clear that some distribution-based methods have been merely focused on minimally detectable changes. For assessing minimally important changes, anchor-based methods are preferred, as they include a definition of what is minimally important. Acknowledging the distinction between minimally detectable and minimally important changes is useful, not only to avoid confusion among MIC methods, but also to gain information on two important benchmarks on the scale of a health status measurement instrument. Appreciating the distinction, it becomes possible to judge whether the minimally detectable change of a measurement instrument is sufficiently small to detect minimally important changes.

## Introduction

Health status questionnaires are increasingly used in medical research and clinical practice. They are attractive because they provide a self-report of patients' perceived health status. However, the meaning of the (changes in) scores on these questionnaires is not intuitively apparent. The interpretation of (change)scores has been a topic of research for almost two decades [1,2]. It is recognized that the statistical significance of a treatment effect, because of its partial dependency on sample size, does not always correspond to the clinical relevance of the effect. Statistically significant effects are those that occur beyond some level of chance. In contrast, clinical relevance refers to the benefits derived from that treatment, its impact upon the patient, and its implications for clinical management of the patient [2,3]. As a yardstick for clinical relevance one is interested in the minimally important change (MIC) of health status questionnaires. Changes in scores exceeding the MIC are clinically relevant by definition.

Different methods to determine the MIC on the scale of a measurement instrument have been proposed. These methods have been summarized by Lydick and Epstein [4], and recently more extensively by Crosby et al. [5]. Both overviews distinguish distribution-based and anchor-based methods [4,5].

Distribution-based approaches are based on statistical characteristics of the sample at issue. Most distribution-based methods express the observed change in a standardized metric. Examples are the effect size (ES) and the standardized response mean (SRM), where the numerators of both parameters represent the mean change and the denominators are the standard deviation at baseline and the standard deviation of change for the sample at issue, respectively. Another distribution-based measure is the standard error of measurement (SEM), which links the reliability of the measurement instrument to the standard deviation of the population [5]. ES and SRM are relative representations of change (without units), whereas the SEM provides a number in the same units as the original measurement. The major disadvantage of all distribution-based methods is that they do not, in themselves, provide a good indication of the importance of the observed change.

Anchor-based methods assess which changes on the measurement instrument correspond with a minimal important change defined on the anchor [4], i.e. an external criterion is used to operationalize a relevant or an important change. The advantage is that the concept of 'minimal importance' is explicitly defined and incorporated in these methods. A limitation of anchor-based approaches is that they do not, in themselves, take measurement precision into account [4,5]. Thus, there is no information on whether an important change according to an anchor-based method, lies within the measurement error of the health status measurement.

An often used anchor-based method is the one proposed by Jaeschke et al. [2], which defined MIC as the mean change in scores of patients categorized by the anchor as having experienced minimally important improvement or minimally important deterioration. Another anchor-based method, proposed by Deyo and Centor [6], is based on diagnostic test methodology. In this method, the change score on the measurement instrument is considered the diagnostic test and the anchor, dividing the population in persons who are minimally importantly changed and those who are not, is considered the gold standard. At different cut-off values of change scores the sensitivity and specificity are calculated and the MIC value is set at the change value on the measurement, where the sum of the percentages of false positives and false negatives is minimal.

A number of studies have compared anchor-based and distribution-based approaches. Comparisons of these approaches sometimes led to surprisingly similar results. However, in other situations different results were found. The focus of this paper is on explanation of the differences between distribution-based and anchor-based approaches. We will provide arguments for the distinction between minimally detectable change and minimally important change. Appreciating and acknowledging this distinction enhances the interpretation of change scores of a measurement instrument.

### Comparison of SEM with anchor-based approaches

A number of studies have compared the value of the SEM with the MIC value derived by an anchor-based approach. A SEM value is easy to calculate, based on the standard deviation (SD) of the sample and the reliability of the measurement instrument: in formula: SEM = SD √(1-R). As reliability parameter, test-retest reliability or Cronbach's α can be used. In the latter case, SEM can be calculated based on one measurement and it purely represents the variability of the instrument [7]. Test-retest reliability requires two measurements in a stable population. It represents the temporal stability and is therefore more appropriate than Cronbach's α to use in the context of changes in health status which are based on measurements at two different time points [8]. In classical test theory, SEM has a rather stable value in different populations [6].

Several authors showed that a MIC based on patient's global rating as anchor was close to the value of one SEM [9,11]. Cella et al. [12] also observed similar values for SEM and MIC, using clinical parameters as anchor instead of patients' global rating of change. However, Crosby et al. [13] showed that only for patients with moderate baseline values the anchor-based MIC value more or less equalled the SEM value (with adjustment for regression to the mean). With higher baseline values the MIC became considerably larger than one SEM, while with lower baseline values the MIC became much smaller than one SEM. A recent study [14] compared SEM with anchor-based estimations of minimally important change using crosssectional and longitudinal anchors. No substantial differences were found between these methods, but it should be noted that they only presented anchor-based values when effect sizes were between 0.2 and 0.5 [14].

Wyrwich [15], compared SEM to MIC values determined by an anchor-based approach in two sets of studies which differed on several points. Set A consisted of studies on musculoskeletal disorders like low back pain [16], neck pain [17] and lower extremity disorders [18], while set B included studies on chronic disorders like chronic respiratory disease [10], chronic heart failure [11], and asthma [19]. In addition, set A studies used the ROC method and studies in set B applied mean change as anchor-based method. And they differed with regard to the definition of 'minimal important change' on the anchor. For set A, the MIC corresponded to 2.3 or 2.6 * SEM, for set B, the MIC values were close to 1*SEM.

In summary, it seems that the proposition that one SEM equals the MIC is not a universal truth.

### MIC is a variable concept

#### 1. MIC depends on the definition of 'important change' on the anchor

A patient's self report global rating scale of perceived change has often been used as anchor. Studies determining MIC have used different definitions of 'minimally importance' using this anchor. Wyrwich et al. [10,11,19] defined a slight change on the anchor as 'minimally important', consisting of the categories "a little worse/better" and "somewhat worse/better". In their earlier studies Wyrwich et al. even included the category "almost the same, hardly any worse/better" [10,11]. Other authors have defined 'minimal importance' as a larger change on the anchor. Binkley et al. [18] chose the category "moderate improvement" as minimally important. Stratford et al. [17] chose to lay the cut-off point for MIC between "moderate" and "a good deal" of improvement. Others [20-24] have laid the cut-off point for MIC between "slightly improved" and "much improved" on the patient global rating scale. In studies requiring moderate or much improvement, the MIC corresponds to about 2.5 times the SEM value. The differences in set A and B in Wyrwich's study [15] may be partly explained by a different definition of important change on the anchor: set A consists of studies which defined MIC as a good deal better [16] and studies in set B [10,11,19] defined MIC as a little and somewhat better according to the anchor.

The MIC value depends to a great degree on the anchor's definition of *minimal importance*. So, the crucial question, then, is "what is a minimally important improvement or deterioration?" Some authors tend to emphasize *minimal*, while others stress *important *[25]. Remarkably, the reference standard is usually based on the amount of change and little research has focused on the "importance" of the change.

#### 2. MIC depends on the type of anchor

Clinicians may have other opinions about what is important than patients. Therefore, clinician-based anchors may lead to different MIC values. Kosinski et al. [26] used five different anchors to estimate the minimally important differences for the SF-36 in a clinical trial of people with rheumatoid arthritis, and found different MIC values dependent on the anchor used. Some authors [16,20-24] have asked patients' global rating of perceived change in overall health, while others asked to rate the perceived change separately for each dimension of their measurement instrument [10,19]. For example, in a study determining the MIC of the Chronic Respiratory Disease Scale the patients' global rating has been asked separately for the subscales dyspnoea, fatigue and emotional function [10]. In the rating of change in overall health status patients have to weigh the relative contribution of the different dimensions on their health status. For example, if patients with asthma judge dyspnoea to be much more important for their quality of life than emotional functioning, a small change in dyspnoea will affect the global rating of overall health, while for emotional functioning the change must be larger to be influential. The observed MIC value will be smallest for the anchor that shows the highest correlation with the health status scale under study.

#### 3. MIC depends on baseline values and direction of change

Several studies have shown that the MIC value of a measurement instrument depends on the baseline score on that instrument. This was clearly shown by Crosby et al. [13] who compared the SEM, corrected for regression to the mean, with the anchor-based MIC for various baseline scores of obesity-specific health related quality of life. With higher baseline values MIC became considerably larger than one SEM. Other authors [16,24,27,28] showed that the values of anchor-based MIC for functional status questionnaires in patients with low back pain were dependent on baseline values. Patients with a high level of functional disability at baseline must change more points on the Roland Disability Questionnaire than patients with less functional disability at baseline to consider it an important change. In addition, Van der Roer et al. [24] reported different MIC values for acute and chronic low back pain patients.

Furthermore, there has been discussion whether the MIC for improvement is the same as for deterioration [5]. In some studies the same MIC is reported for patients who improve and patients who deteriorate [2,29,30], but others found different MIC values for improvement and deterioration. Cella et al. [31] demonstrated that cancer patients who reported global worsening had considerably larger change scores on the Functional Assessment of Cancer Therapy (FACT) scale than those reporting comparable global improvements. Also Ware et al. observed that a larger change on the SF-36 was needed for patients to feel worsened than to feel improved [32].

Thus, the MIC is dependent on, among other things, the type of anchor, the definition of 'minimal importance' on the anchor, and on the baseline score which might be an indicator of severity of the disease. Therefore, various authors have suggested to present a range of MIC values [24,26,33-35], to account for this diversity. Hays et al. recommend to use different anchors and to give reasonable bounds around the MIC, rather than forcing the MIC to be a fixed value [33,34].

### Distinction between minimally detectable and minimally important changes

Some authors have searched for uniform measures for minimally important changes. Wyrwich and others [10,11] have evaluated whether the one-SEM criterion can be applied as a proxy for MIC. Norman et al. [36] made a systematic review of 38 studies (including 62 effect sizes), and observed, with only a few exceptions, that the MICs for health related quality of life instruments were close to half a standard deviation (SD). This held for generic and disease specific measures and was not dependent on the number of response options.

Norman et al. [36] explain their finding of 0.5 SD by referring to psychophysiological evidence that the limit of people's ability to discriminate is approximately 1 part in 7, which is very close to half a SD. Thus, this criterion of 0.5 SD may be considered a threshold of detection and corresponds more to *minimally detectable *change than to *minimally important *change. Also SEM, based on the test-retest reliability in stable persons, is merely a measure of detectable change [37]. Note that, using the formula SEM = SD √(1-R), 1 SEM equals 0.5 SD, when the reliability of the instrument is 0.75. Thus, 0.5 SD and SEM clearly alert to the concept of minimally detectable changes.

Wyrwich [15] comparing the two sets of studies showed that if the cut-off point for 'minimal importance' on the anchor is laid between "no change" and "slightly changed", i.e. the first category above no change, together with a complaint-specific anchor, the MIC is close to one SEM. But in this case it focuses more on *minimally detectable *change than *minimally important *change. Wyrwich [15] showed a clear dependency between MIC and cut-off value of 'minimal importance' on the anchor of patients' global rating of perceived change.

Salaffi et al. [38] presented the change on a numerical rating scale for pain using two cut-off points on a patient global impression of change scale. In their opinion, a MIC using "slightly better" as cut-off point on the anchor reflected the minimum and lowest degree of improvement that could be detected, while the cut-off point "much better" refers to a clinically important outcome. Note that the choice of anchor and cut-off point is arbitrary and cannot be based on statistical characteristics.

### Interpretation and applicability

We believe that the confusion about MIC will decrease if the distinction between minimally detectable and minimal important change is appreciated and acknowledged.

In statistical terms, the minimally detectable change (MDC), also called smallest detectable change or smallest real change [37] shows which changes fall outside the measurement error of the health status measurement (either based on internal or test-retest reliability in stable persons). It is represented by the following formula: MDC = 1.96 * √2 * SEM, where the 1.96 derives from the 95% confidence interval of no change, and √2 is included because two measurements are involved in measuring change [37].

As a different concept, the MIC value depicts changes which are considered to be minimally important by patients, clinicians, or relevant others. The SEM, the minimally detectable change and the minimally important change are all important benchmarks on the scale of the measurement instrument, which helps with the interpretation of change scores.

Appreciating the distinction, we can answer the important question whether a health status measurement instrument is able to detect changes as small as the MIC value. This application is shown in a study on measurement instruments for low back pain [27] and for visual impairments [39].

## Conclusion

Some distribution-based methods to assess MIC have been more focussed on minimally detectable changes than on minimally important changes. For assessing minimally important changes, anchor-based methods are preferred, as they include a definition of what is minimally important. Acknowledging the distinction between minimally detectable and minimally important changes is useful, not only to avoid confusion among MIC methods, but also to gain information on two important benchmarks on the scale of a health status measurement instrument. Moreover, it becomes possible to judge whether the minimally detectable change of a measurement instrument is sufficiently small to detect minimally important changes.
